# Correction: A tripartite bacterial-fungal-plant symbiosis in the mycorrhiza-shaped microbiome drives plant growth and mycorrhization

**DOI:** 10.1186/s40168-024-01776-2

**Published:** 2024-02-19

**Authors:** Changfeng Zhang, Marcel G. A. van der Heijden, Bethany K. Dodds, Thi Bich Nguyen, Jelle Spooren, Alain Valzano‑Held, Marco Cosme, Roeland L. Berendsen

**Affiliations:** 1 Plant‑Microbe Interactions, Department of Biology, Faculty of Science, Utrecht University, Padualaan 8, 3584 CH Utrecht, the Netherlands; 2 Plant Soil Interactions, Division Agroecology and Environment, Agroscope, Reckenholzstrasse 191, CH‑8046 Zürich, Switzerland; 3 Department of Plant and Microbial Biology, University of Zurich, Zollikerstrasse 107, CH‑8008 Zurich, Switzerland; 4 Mycology, Earth and Life Institute, Université Catholique de Louvain, Louvain‑La‑Neuve, Belgium; 5 Plants and Ecosystems, Biology Department, University of Antwerp, Antwerp, Belgium

**Correction** **:** **Microbiome 12, 13 (2024)**

**https://doi.org/10.1186/s40168-023-01726-4**

Following publication of the original article [1], it was found that there were typographical errors in Figures 5-8. The term “AMFungal” should be “AM Fungal” and “colornization” should be “colonization” in those figures.

The incorrect figures are:

**Fig. 5:**
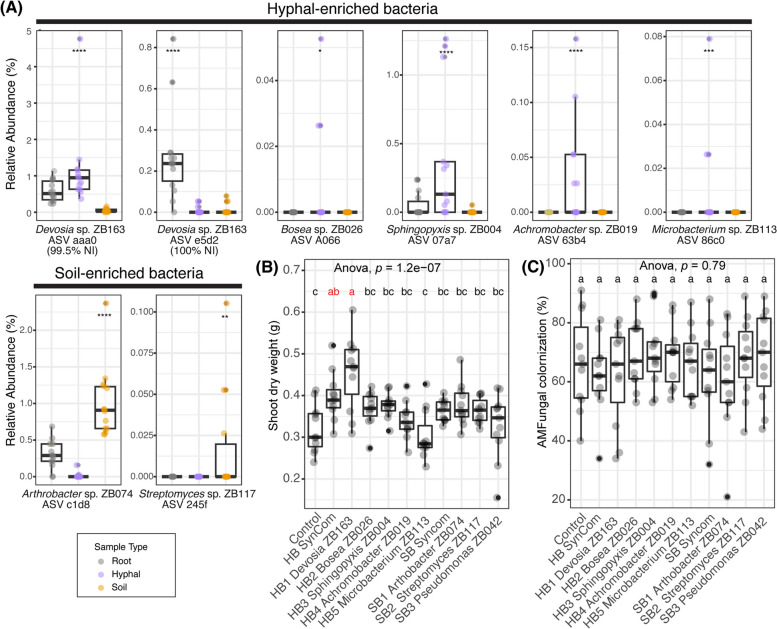
*Devosi*a sp. ZB163 is isolated from fungal hyphae but thrives on the root and promotes plant growth. **A** Relative abundance of the selected ASVs in the root, hyphal, and soil samples in Experiment I. Sample types were indicated by color. Each selected ASVs ID was labeled together with a selected corresponding bacterial isolate with matching sequence. The significance levels, as determined by *Indicspecies*, for the ASVs exhibiting positive correlations with hyphal (ASV aaa0, A066,0,7a7, 63b4 and 86c0) root (e5d2), or soil (c1d8 and 254f ) samples are denoted by asterisks (*p < 0.05, **p < 0.01, ***p < 0.001, ****p < 0.0001). **B** Shoot dry weight of 9-week-old Prunella plants (**C**) AM fungi colonization percentage comparison between bacterial treatments. Significant differences of (**B**) and (**C**) are indicated with letters (ANOVA and Tukey’s Honest HSD test)

**Fig. 6:**
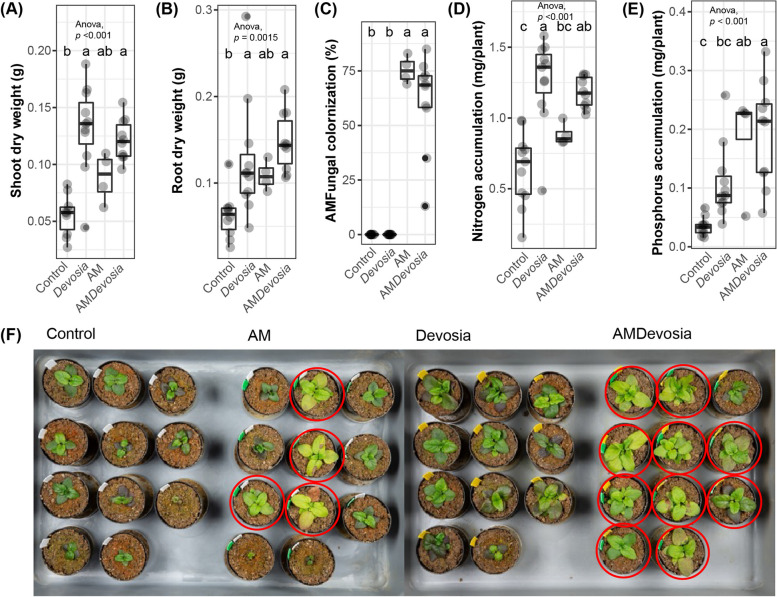
*Devosia* promotes plant growth, mycorrhization, and N accumulation. Boxplots show **A** shoot dry weight, **B** root dry weight, **C** percentage of each root system colonized by AM fungi, **D** shoot N accumulation, and **E** shoot P accumulation of 8-week-old Prunella plants cultivated in autoclaved soil (Control) or inoculated with *Devosia* sp. ZB163 (Devosia), *R. irregularis* (AM), or both symbionts. In the 6th, 7th and 8th week, plants were watered with modified Hoagland solution without N and P. Significant differences are indicated with letters (ANOVA and Tukey’s Honest HSD test). **F** Photographs of the Prunella plants immediately before harvest. Red circles indicate plants that were later found to be colonized by AM fungi

**Fig. 7:**
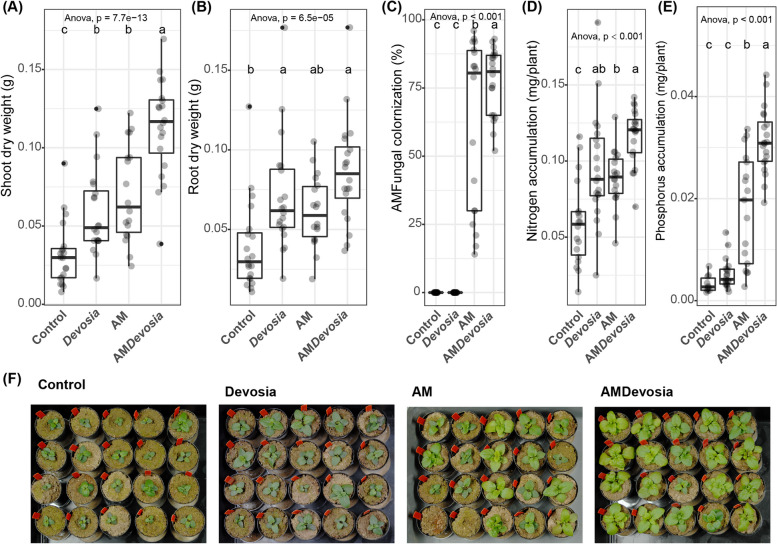
*Devosia* sp. ZB163 and AM fungi can synergistically promote plant growth and plant N and P accumulation. Boxplots show **A** shoot dry weight, **B** root dry weight, **C** percentage of each root system colonized by AM fungi, **D** shoot N accumulation, or **E** shoot P accumulation of 8-week-old Prunella plants cultivated in autoclaved soil (Control) or inoculated with *Devosia* sp. ZB163 (Devosia), *R. irregularis* (AM), or both symbionts. Plants were regularly watered with modified Hoagland solution deficient in a source of N and P. Significance differences are indicated with letters (ANOVA and Tukey’s Honest HSD test). **F** Photographs of the Prunella plants immediately before harvest. Two AM-treated plants died shortly after transplantation and were not considered in panels (**A**–**E**)

**Fig. 8:**
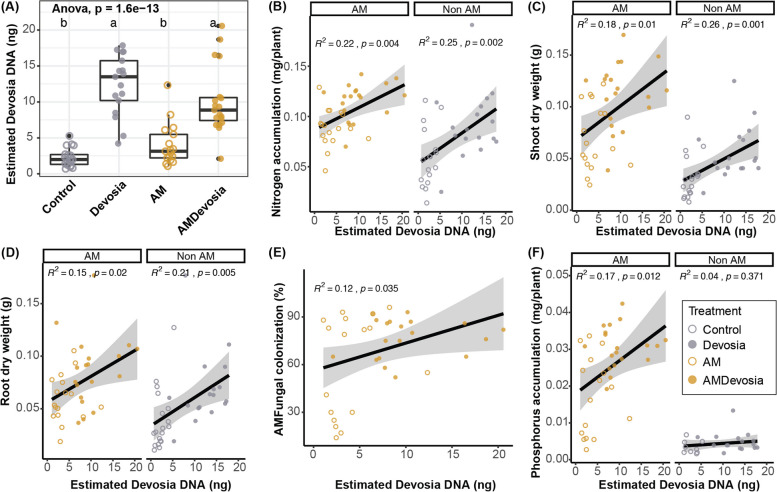
Abundance of *Devosia* sp. ZB163 significantly correlates with plant weight, mycorrhization, and N and P accumulation. **A** Boxplot of the absolute abundance of *Devosia* DNA on roots of plants in sterilized soil inoculated with a mock solution (Control), *Devosia* sp. ZB163 (*Devosia*), *R. irregularis* (AM), or both symbionts. Letters indicate significant differences as determined by ANOVA with Tukey’s HSD test. **B**–**E** Scatter plots of the correlation between the absolute abundance of *Devosia* DNA and **B** total plant N accumulation, **C** shoot dry weight, **D** root dry weight, **E** hyphal colonization, and **F** total plant P accumulation. Correlations and probabilities thereof are determined using linear regression

The correct figures are:

**Fig. 5:**
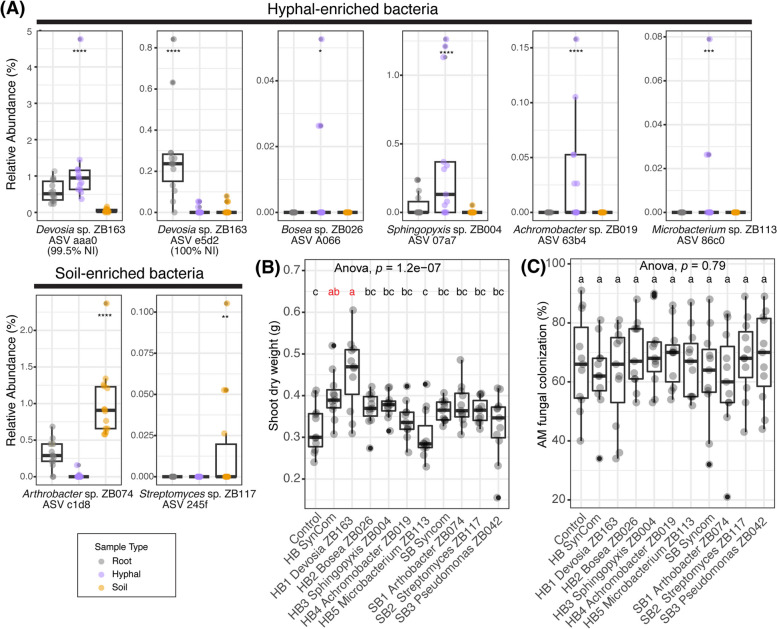
*Devosi*a sp. ZB163 is isolated from fungal hyphae but thrives on the root and promotes plant growth. **A** Relative abundance of the selected ASVs in the root, hyphal, and soil samples in Experiment I. Sample types were indicated by color. Each selected ASVs ID was labeled together with a selected corresponding bacterial isolate with matching sequence. The significance levels, as determined by *Indicspecies*, for the ASVs exhibiting positive correlations with hyphal (ASV aaa0, A066,0,7a7, 63b4 and 86c0) root (e5d2), or soil (c1d8 and 254f ) samples are denoted by asterisks (*p < 0.05, **p < 0.01, ***p < 0.001, ****p < 0.0001). **B** Shoot dry weight of 9-week-old Prunella plants (**C**) AM fungi colonization percentage comparison between bacterial treatments. Significant differences of (**B**) and (**C**) are indicated with letters (ANOVA and Tukey’s Honest HSD test)

**Fig. 6:**
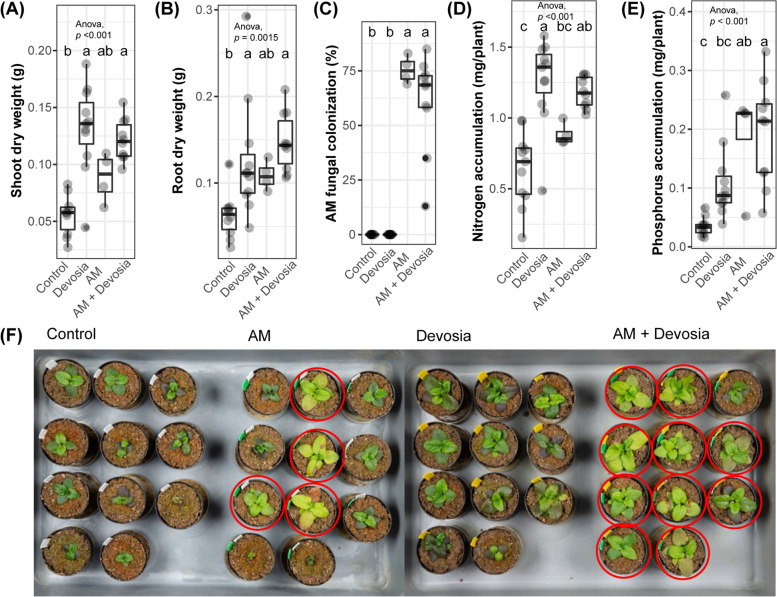
*Devosia* promotes plant growth, mycorrhization, and N accumulation. Boxplots show **A** shoot dry weight, **B** root dry weight, **C** percentage of each root system colonized by AM fungi, **D** shoot N accumulation, and **E** shoot P accumulation of 8-week-old Prunella plants cultivated in autoclaved soil (Control) or inoculated with *Devosia* sp. ZB163 (Devosia), *R. irregularis* (AM), or both symbionts. In the 6th, 7th and 8th week, plants were watered with modified Hoagland solution without N and P. Significant differences are indicated with letters (ANOVA and Tukey’s Honest HSD test). **F** Photographs of the Prunella plants immediately before harvest. Red circles indicate plants that were later found to be colonized by AM fungi

**Fig. 7:**
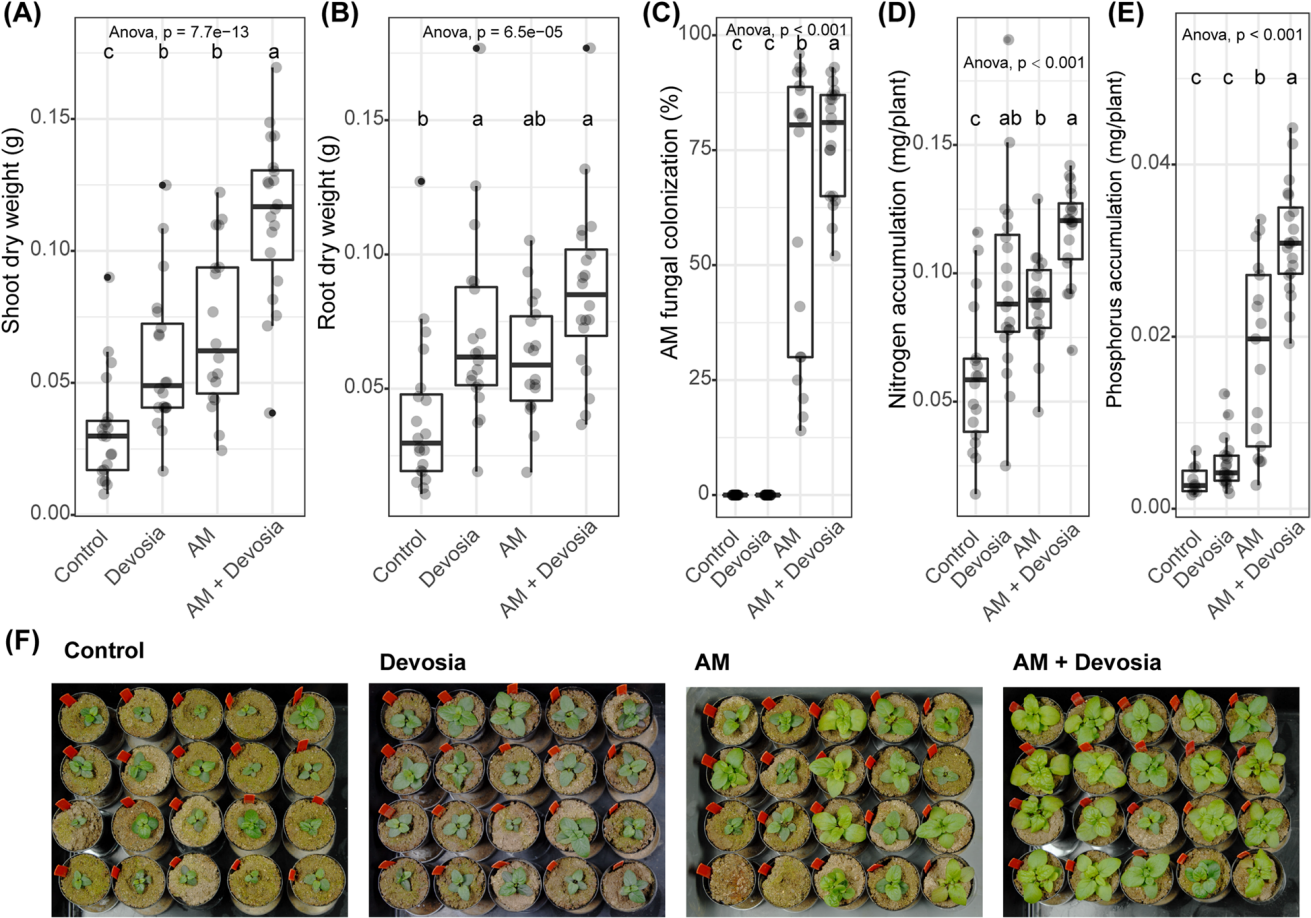
*Devosia* sp. ZB163 and AM fungi can synergistically promote plant growth and plant N and P accumulation. Boxplots show **A** shoot dry weight, **B** root dry weight, **C** percentage of each root system colonized by AM fungi, **D** shoot N accumulation, or **E** shoot P accumulation of 8-week-old Prunella plants cultivated in autoclaved soil (Control) or inoculated with *Devosia* sp. ZB163 (Devosia), *R. irregularis* (AM), or both symbionts. Plants were regularly watered with modified Hoagland solution deficient in a source of N and P. Significance differences are indicated with letters (ANOVA and Tukey’s Honest HSD test). **F** Photographs of the Prunella plants immediately before harvest. Two AM-treated plants died shortly after transplantation and were not considered in panels (**A**–**E**)

**Fig. 8:**
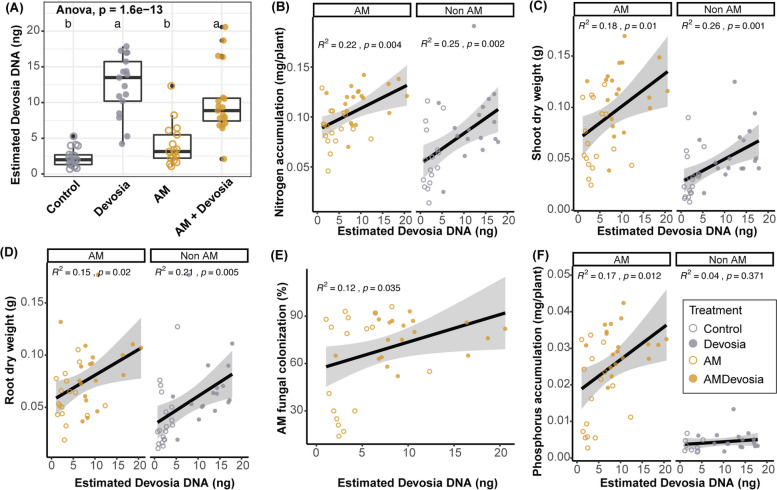
Abundance of *Devosia* sp. ZB163 significantly correlates with plant weight, mycorrhization, and N and P accumulation. **A** Boxplot of the absolute abundance of *Devosia* DNA on roots of plants in sterilized soil inoculated with a mock solution (Control), *Devosia* sp. ZB163 (*Devosia*), *R. irregularis* (AM), or both symbionts. Letters indicate significant differences as determined by ANOVA with Tukey’s HSD test. **B**–**E** Scatter plots of the correlation between the absolute abundance of *Devosia* DNA and **B** total plant N accumulation, **C** shoot dry weight, **D** root dry weight, **E** hyphal colonization, and **F** total plant P accumulation. Correlations and probabilities thereof are determined using linear regression

The original article has been updated  to correct Figures 5-8.
